# Artificial Aging and Its Impact on Surface Properties of Conventional and CAD–CAM Occlusal Splint Materials: An In Vitro Comparative Study

**DOI:** 10.1155/ijod/3840775

**Published:** 2026-09-09

**Authors:** Uthai Uma, Jirayu Chansri, Thitiphat Itthipornpaisan, Trin Thahong

**Affiliations:** ^1^ Department of Occlusion, Faculty of Dentistry, Chulalongkorn University, 34 Henri-Dunant Road, Wangmai, Pathumwan, Bangkok 10330, Thailand, chula.ac.th; ^2^ Faculty of Dentistry, Chulalongkorn University, 34 Henri-Dunant Road, Wangmai, Pathumwan, Bangkok 10330, Thailand, chula.ac.th

**Keywords:** CAD–CAM, color stability, dental materials, dental technology, microhardness, occlusal splint, surface roughness

## Abstract

**Purpose:**

This study aimed to evaluate and compare the surface roughness, microhardness, and color stability of various materials after dry storage (control) and artificial aging (intervention).

**Materials and Methods:**

One‐hundred‐eight 10 × 10 × 5 mm^3^ specimens were fabricated from three clear acrylic resin types: heat‐cured (HC), milled (ML), and 3D‐printed (3D). Each specimen underwent a stepwise polishing process on one side. For the pre‐test measurement, 36 specimens (12 per group) were evaluated for surface roughness (Ra), 36 for Vickers microhardness (Vickers hardness number [VHN]), and 36 for color stability (∆*E*). Half of the specimens in each test were stored in dry conditions for 30 days, while the other half was aged in 37°C distilled water for 30 days. Post‐test evaluations were conducted using the same procedures. The statistical analyses of Ra, VHN, and ∆*E* were performed using SPSS 29.0.

**Results:**

Significant differences in Ra were observed among all materials (*p* < 0.05), with ML and 3D exhibiting low Ra. The artificial aging condition did not significantly influence Ra, however, polishing significantly affected Ra. The VHN values also differed significantly among materials (*p* < 0.05), with ML demonstrating the highest VHN. Polishing did not affect VHN, but all materials had reductions in VHN (*p* = 0.002) after artificial aging. The ∆*E* values varied significantly across material types, with ML demonstrating the least color change.

**Conclusion:**

This study demonstrated significant variations in surface roughness, microhardness, and color stability among occlusal splint materials after the aging conditions. ML exhibited superior performance with low Ra, low ∆*E*, and high VHN.

## 1. Introduction

Temporomandibular disorders (TMD) and bruxism can be managed using a variety of conservative treatment approaches, including patient education, self‐management, behavioral therapy, physiotherapy, counseling, relaxation and breathing exercises, pharmacological therapy, occlusal splint therapy, and other reversible interventions [1, 2]. Occlusal splints are removable intraoral appliances designed to fit over the dental arches and are widely prescribed for the management of TMD and bruxism [3, 4]. In recent years, advances in digital technologies and manufacturing processes have led to the development of new fabrication methods for occlusal splints, providing clinicians with a broader range of material and production options [5].

Occlusal splints can be manufactured by various methods, such as the sprinkle‐on, lost‐wax, thermoforming, additive (3D‐printing), and subtractive (milling) techniques [5]. However, these methods are typically categorized into two main types based on the overall manufacturing process. The analog workflow requires oral impressions to be taken by dentists, with potential errors possible in each fabrication step. In contrast, the digital workflow employs innovative technologies and advanced dental equipment to produce the oral appliance using a combination of computer‐aided design (CAD) and computer‐aided manufacturing (CAM) [6, 7]. Each workflow has its own advantages and disadvantages when considering suitable methods for dental patients. Furthermore, the dental materials, especially polymethyl methacrylate (PMMA) used in different manufacturing technique [8], vary in their properties [5], which should be evaluated before being prescribed to patients.

Numerous studies examined and reported on the mechanical properties of occlusal splints, including surface roughness [9, 10], surface hardness [10–12], flexural strength [11, 12], modulus of elasticity [11, 12], compression and tensile tests [13], degree of conversion [12], wear resistance [14], water sorption [15], water solubility [15], and chemical stability [16]. These mechanical properties should be evaluated under thermal and/or mechanical artificial aging conditions to determine whether occlusal splints fabricated using conventional or digital workflows are suitable for clinical use [17, 18].

Although the techniques for investigating the properties of occlusal splints are similar, there remains a lack of knowledge in many aspects that require further study. In particular, understanding which fabrication techniques produce occlusal splint materials with the best surface roughness, microhardness, and color stability is crucial. Moreover, the differences in properties between polished and unpolished surfaces on conventional and digital occlusal splints are not yet clear. Hence, the aim of this study was to investigate and compare the surface roughness, microhardness, and color stability among three groups of occlusal splint materials made by heat‐curing, milling, and 3D‐printing fabrication techniques with unpolished and polished sides. The null hypothesis stated that there would be no significant differences in any of the evaluated properties after dry storage (control) and artificial aging (intervention) among the three material groups.

## 2. Materials and Methods

**Figure 1:**
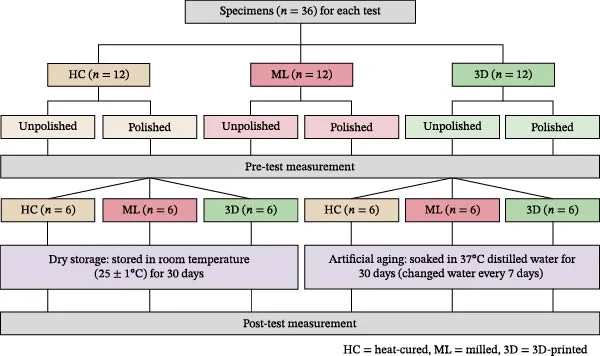
Study design illustrating the allocation of specimens for each test. A total of 108 specimens were prepared and equally divided among three tests: surface roughness, Vickers microhardness, and color stability (*n* = 36 per test). For each test, specimens were further categorized into three occlusal splint material groups: heat‐cured (HC), milled (ML), and 3D‐printed (3D).

The specimens were prepared for three laboratory tests comprising surface roughness, microhardness, and color stability, as shown in Figure 1. The independent variable of this study was the three types of occlusal splint materials fabricated by different techniques [10]. The study determined the mean differences in the evaluated parameters among the three specimen groups. As this investigation was an in vitro laboratory study that did not involve human participants, human tissues, biological specimens, or identifiable personal data, ethical approval was not required.

The sample size was estimated using G^ ∗^Power software version 3.1.9.7, based on the *F*‐test family and one‐way ANOVA. According to Uma et al. [10], the mean surface roughness values of occlusal splint materials, based on 12 specimens per group, were 0.064 ± 0.016, 0.046 ± 0.009, and 0.082 ± 0.019 µm. Based on these values, the calculated effect size was 0.8645. The statistical parameters were set at 80% power, a significance level of 5%, and three material groups. The minimum required sample size was, therefore, six specimens per material group for each test.

Two aging conditions were included: dry storage and immersion in distilled water at 37°C for 30 days. Accordingly, the sample size was doubled to 12 specimens per material group for each test. With three material groups, a total of 36 specimens were required for each test. Three surface properties were evaluated: surface roughness, microhardness, and color stability. Therefore, the total number of specimens required for the study was 108 (Figure 1).

### 2.1. Specimen Preparation

The specimen dimensions were standardized across all experimental groups, including heat‐cured (HC), milled (ML), and 3D‐printed occlusal splint materials. A specimen size of 10 × 10 × 5 mm^3^ was selected based on dimensions used in previous studies [10, 19, 20].

Group 1: HC occlusal splint: Specimens were created using 10 × 10 × 5 mm^3^ rectangular wax patterns that were placed in flasks, and the wax was melted out and replaced with mixed clear acrylic resin powder and monomer (Meliodent Heat Cure, Kulzer GmbH, Hanau, Germany), following the manufacturer’s instruction with a ratio of 35 g:14 mL (powder:liquid). A heat‐curing process was applied by placing the flask in boiling water for 20 min to achieve complete polymerization, as recommended by the manufacturer (Table 1).

**Table 1:** Types of occlusal splint materials and their fabrication techniques.

Group	Abbreviation	Material composition	Fabrication techniques
Heat‐cured	HC	Meliodent heat cure: clear (liquid: methyl methacrylate, 1,4‐butandioldimethacrylate, p‐mentha‐1,4‐diene), (powder: polymethyl methacrylate)	Mixed powder and monomer, polymerized in boiling water for 20 min
Milled	ML	Smile‐Cam PMMA calcinable: transparent (polymethyl methacrylate)	Fabricated using a 5‐axis milling machine
3D‐printed	3D	MED610, biocompatible clear (isobornyl acrylate, acrylic monomer, urethane acrylate, acrylic monomer, epoxy acrylate, acrylate oligomer, photoinitiator)	Printed using a 3D printer and post‐cured in a UV light box for 30 min

Group 2: ML occlusal splint: The dimensions were determined in CAD software (Blender 2.79, Blender Institute BV, Amsterdam, Netherlands) to achieve the 10 × 10 × 5 mm^3^ target size. A clear PMMA block (Smile‐Cam PMMA Calcinable, Pressing Dental SRL, Dogana, San Marino Republic) was then ML into the desired dimensions using a 5‐axis milling machine (vnf S2, vhf camfacture AG, Germany) (Table 1).

Group 3: 3D‐printed (3D) occlusal splint: The desired specimen dimensions (10 × 10 × 5 mm^3^) were created using CAD software as Group 2 and then printed using clear resin (MED610, Biocompatible Clear, Stratasys, Minnesota, USA) with a 3D printer (Stratasys Objet260 Dental, USA). The printer used polyJet technology to simultaneously deposit multiple photopolymer materials in ultrafine layers, which were immediately cured with ultraviolet (UV) light. The specimens were printed using the standard printing parameters provided by the manufacturer, including 0‐degree orientation, 0.1‐mm layer thickness, high speed, high quality, and digital material printing mode. Subsequently, the specimens were cleaned in an ultrasonic bath containing isopropyl alcohol for 5 min to eliminate the uncured resin, followed by rinsing with water. They were then post‐cured under UV light (wavelength range: 315–550 nm; peak wavelengths at ∼360 and 435 nm) in a curing box (LC‐3DPrint Box, NextDent, Vertex‐Dental B.V., Soesterberg, the Netherlands) for 30 min (Table 1).

For the polishing procedure, each specimen was designated to have two surfaces: an unpolished surface and a polished surface [20]. The unpolished surface represented the original material surface without any finishing or polishing procedures. For the HC specimens, the surface that did not contact the silicone mold during polymerization was designated as the unpolished surface. For the ML specimens, the unpolished surface was randomly assigned because all specimen surfaces were uniformly prepared by the milling burs. For the 3D specimens, the surface that did not contact the support structures during printing was designated as the unpolished surface. Consequently, the opposing surfaces were designated as the polished surfaces: the mold‐contacting surface for HC specimens, a randomly assigned opposing surface for ML specimens, and the support‐contacting surface for 3D specimens. The designated polished surfaces were finished using waterproof abrasive papers (600‐, 800‐, and 1000‐grit; TOA Paint, Samut Prakan, Thailand) on an automatic polishing machine (EcoMet 30, Buehler, Lake Bluff, IL, USA) at 500 rpm under a 5 N load for 1 min per step. Final polishing was performed with wet pumice (Lab Pumice, Medium Grit, Shanghai Dental Material, Bangkok, Thailand) followed by tallow (Shanghai Dental Material, Bangkok, Thailand) on a polishing lathe (Polix 905, Silfradent, Santa Sofia, Italy) at 1400 rpm for 1 min to achieve a high‐gloss surface. After specimen preparation, all samples were stored in a dry container at room temperature for 7 days prior to testing. This storage period was intended to simulate the transportation and handling time from the dental laboratory to the clinical setting.

### 2.2. Measurement of Surface Properties

For the pre‐test, one‐third of the specimens (*n* = 36) were measured for roughness using a contact surface roughness tester (Stylus for contact, Talyscan 150, England) equipped with a stylus, following a protocol adapted from ISO 21920. Each specimen was tested three times at random areas around the center, using a contact length of 5 mm per time, and the average surface roughness value (Ra) was used for further analysis.

Additionally, one‐third of the specimens (*n* = 36) were tested for Vickers microhardness following protocols adapted from ISO 6507 and ASTM E384. The Vickers surface hardness was measured using a microhardness tester with a Vickers indenter (FM810, Future‐Tech, Japan) with a 1.96 N load applied for 15 s [21]. The diagonals of the pyramid impressed on the specimen were optically measured on a microscopic scale by the eyepiece operator of the testing machine, and the Vickers hardness number (VHN) was calculated using the formula, VHN = 0.1891(*F*/*d* ^2^), where *F* is the force in newtons and *d* is the mean length of the two diagonals in millimeters. Each specimen was measured for Vickers microhardness three times in different areas, and the average value was calculated for further analysis.

The remaining one‐third of the specimens (*n* = 36) were tested for color stability using a spectrocolorimeter (Ultrascan PRO, Hunter Lab, USA) with the reflectance mode and a small area view (7 mm port size and 4 mm measured area), following a protocol adapted from ISO 7491, using the CIELAB system according to ISO 11664–4:2008. The measurement was performed three times on different areas, and the average color stability value (∆*E*) was calculated from the formula, ∆*E* = ([∆*L*]^2^ + [∆*a*]^2^ + [∆*b*]^2^)^1/2^, where *L* represents lightness, *a* represents the redness–grayness of the color, and *b* denotes the yellow‐blueness of the color [6, 7].

For the aging intervention, half of the specimens in each test group were stored under dry conditions at room temperature (25 ± 1°C) for 30 days (Figure 1), while the remaining half were immersed in distilled water at 37°C using a water bath (Single Hole BHS‐1, Joanlab, Zhejiang, China). This artificial aging protocol was adapted from previous studies [12, 21, 22]. The distilled water was replaced every 7 days throughout the aging period. Water immersion at 37°C was used to simulate the moisture exposure associated with the intraoral use of occlusal splints. However, this aging protocol did not reproduce the complexity of the oral environment, including the effects of saliva, pH fluctuations, enzymatic activity, thermal cycling, cyclic mechanical loading, toothbrushing, disinfection agents, dietary staining, cleaning solutions, and parafunctional loading. Although no standardized artificial aging protocol has been established for occlusal splint materials [22], the selected 30‐day water immersion period was intended to approximate 90 nights of clinical use, assuming nocturnal wear of about 8 h per night. Finally, the specimens were removed from the water, gently dried, and conditioned at room temperature (25 ± 1°C) for at least 48 h before the next measurements.

For the post‐test, the surface roughness, microhardness, and color stability tests were performed using the same protocols as for the pre‐test. Additionally, one specimen per group, totaling six specimens, was randomly selected to be sputter‐coated with a 99.99% gold layer in a vacuum evaporator using a sputter coater (Gold Coater, JFC‐1200, Jeol, USA) to examine the surface roughness using scanning electron microscopy (SEM) (Quanta 250 FEG, FEI, USA), operating in high vacuum mode at a magnification of 1000×.

### 2.3. Data Collection and Statistical Analysis

To ensure consistent data collection standards, a single examiner was selected for each laboratory test to measure and record the pre‐ and post‐test data for the specimens. Statistical analysis was performed using IBM SPSS Statistics for Windows, Version 29.0 (IBM Corporation, Armonk, New York, USA). The Shapiro–Wilk test assessed the normal distribution of the data. One‐way ANOVA was used to compare the mean differences among the three groups of materials. The paired *t*‐test compared the pre‐ and post‐test results within each group. The independent *t*‐test compared the mean differences between the polished and unpolished sides and between dry storage and artificial aging. Two‐way ANOVA evaluated the factors affecting changes in occlusal splint material properties. Mean differences were considered significant at a 95% confidence level with a *p*‐value of 0.05.

## 3. Results

The data presented in Table 2 show the mean values of surface roughness (Ra, µm), Vickers microhardness (VHN, N/mm^2^), and color stability (Δ*E*) for the three types of occlusal splint materials. Measurements were recorded at baseline (pre‐test) and after aging (post‐test), including dry storage and immersion in distilled water at 37°C for 30 days. The differences between post‐ and pre‐test values are also presented.

**Table 2:** Mean values of surface roughness (Ra, µm), Vickers microhardness (VHN, N/mm^2^), and color stability (Δ*E*) of the three types of occlusal splint materials under different aging conditions (mean ± SD).

Surface properties		Unpolished sides			Polished sides		
		HC (*n* = 6)	ML (*n* = 6)	3D (*n* = 6)	HC (*n* = 6)	ML (*n* = 6)	3D (*n* = 6)
Surface roughness (Ra, µm)							
Dry storage	Ra_(pre-test)_	0.282 ± 0.042	0.090 ± 0.028	0.038 ± 0.001	0.061 ± 0.010	0.046 ± 0.010	0.082 ± 0.010
	Ra_(post-test)_	0.262 ± 0.041	0.082 ± 0.015	0.035 ± 0.003	0.066 ± 0.020	0.044 ± 0.010	0.073 ± 0.017
	ΔRa	−0.020 ± 0.025	−0.008 ± 0.004	−0.004 ± 0.003	0.005 ± 0.020	−0.002 ± 0.015	−0.009 ± 0.008
Artificial aging	Ra_(pre-test)_	0.293 ± 0.042	0.090 ± 0.020	0.037 ± 0.002	0.067 ± 0.020	0.047 ± 0.009	0.082 ± 0.020
	Ra_(post-test)_	0.267 ± 0.036	0.092 ± 0.026	0.035 ± 0.001	0.066 ± 0.024	0.050 ± 0.009	0.092 ± 0.030
	ΔRa	−0.026 ± 0.067	0.002 ± 0.020	−0.002 ± 0.002	−0.001 ± 0.030	0.003 ± 0.012	0.009 ± 0.024

Vickers microhardness (VHN, N/mm^2^)							
Dry storage	VHN_(pre-test)_	13.61 ± 1.30	17.03 ± 1.24	12.25 ± 1.09	13.84 ± 0.75	17.27 ± 0.84	12.41 ± 0.43
	VHN_(post-test)_	12.97 ± 0.41	14.43 ± 1.14	11.20 ± 1.07	12.85 ± 0.48	13.99 ± 1.00	11.09 ± 0.71
	ΔVHN	−0.64 ± 1.13	−2.60 ± 1.42	−1.05 ± 1.83	−0.99 ± 0.83	−3.28 ± 1.19	−1.32 ± 0.63
Artificial aging	VHN_(pre-test)_	13.76 ± 0.22	16.73 ± 1.02	12.36 ± 0.95	13.13 ± 1.22	16.75 ± 0.75	12.58 ± 0.30
	VHN_(post-test)_	12.58 ± 0.57	13.76 ± 0.92	9.92 ± 0.38	13.08 ± 0.57	13.73 ± 0.15	9.74 ± 0.47
	ΔVHN	−1.18 ± 0.61	−2.97 ± 0.97	−2.44 ± 0.81	−0.05 ± 1.30	−3.02 ± 0.80	−2.85 ± 0.73

Color stability (Δ*E*)							
Dry storage	Δ*E*	6.22 ± 0.92	3.09 ± 0.86	3.55 ± 0.32	5.79 ± 0.62	3.32 ± 0.25	3.28 ± 0.72
Artificial aging	Δ*E*	10.03 ± 4.76	4.05 ± 0.82	6.79 ± 1.19	8.30 ± 2.04	4.32 ± 0.86	6.52 ± 0.68

*Note*: HC, heat‐cured group; ML, milled group; 3D, 3D‐printed group.

### 3.1. Surface Roughness Analysis

**Figure 2:**
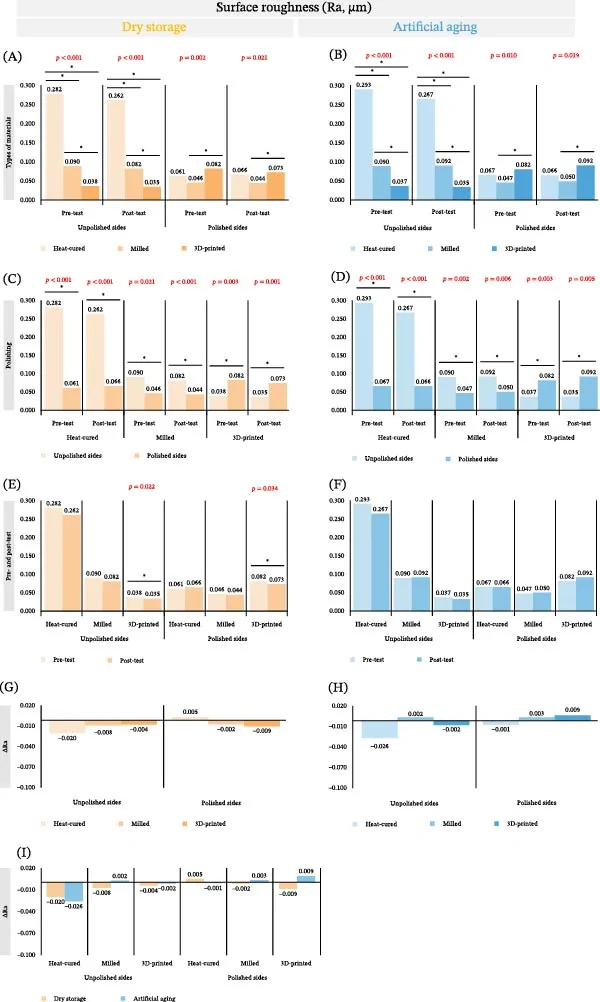
Surface roughness (Ra, µm) of occlusal splint materials under different aging conditions. (A, B) Comparison of surface roughness among heat‐cured, milled, and 3D‐printed materials on unpolished and polished sides under (A) dry storage and (B) artificial aging conditions. (C, D) Effect of polishing on surface roughness within each material type under (C) dry storage and (D) artificial aging. (E, F) Comparison between pre‐test and post‐test surface roughness values for each material and surface condition under (E) dry storage and (F) artificial aging. (G, H) Changes in surface roughness (ΔRa) after testing for each material on unpolished and polished sides under (G) dry storage and (H) artificial aging. (I) Overall comparison of ΔRa between dry storage and artificial aging conditions across all materials and surface treatments. Bars represent mean Ra values (µm). Asterisks ( ^∗^) indicate statistically significant differences (*p* < 0.05), and exact *p*‐values are shown where applicable.

Figure 2 presents the mean surface roughness values of the three types of occlusal splint materials. Significant differences in surface roughness were observed among the materials (*p* < 0.05) at both pre‐test and post‐test under dry storage (Figure 2A) and artificial aging conditions (Figure 2B). The 3D material exhibited the lowest surface roughness on unpolished surfaces, whereas the ML material showed the lowest roughness on polished surfaces. In contrast, the HC material consistently demonstrated the highest surface roughness on both unpolished and polished surfaces (Figure 2A,B).

The mean surface roughness values between unpolished and polished surfaces were further compared (Figure 2C,D). For the HC and ML groups, polishing significantly reduced surface roughness compared with the unpolished surfaces (*p* < 0.05). Conversely, the 3D material showed a significant increase in surface roughness after polishing (*p* < 0.05). Notably, only the unpolished HC specimens exhibited Ra values exceeding the bacterial colonization threshold (Ra > 0.2 µm).

Although post‐test values showed both increases and decreases in surface roughness relative to the baseline, these changes were not statistically significant for most materials (Figure 2E,F). However, the 3D material demonstrated a significant reduction in surface roughness on both unpolished and polished surfaces after dry storage (Figure 2E).

No significant differences in the change in surface roughness (ΔRa) were observed among the three material types under either dry storage or artificial aging conditions (*p* > 0.05) (Figure 2G–I). This finding suggests that the aging conditions did not significantly influence the surface roughness of the occlusal splint materials.

**Figure 3:**
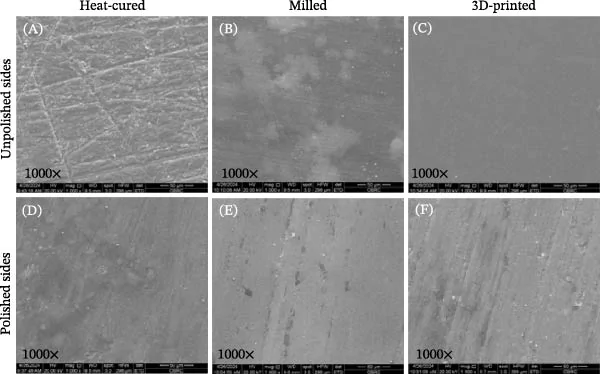
Representative scanning electron microscope (SEM) images (1000× magnification) showing surface features of three occlusal splint materials. Unpolished heat‐cured resin (A) exhibits the roughest surface with deep grooves, while milled (B) and 3D‐printed (C) materials appear progressively smoother. After polishing, the heat‐cured surface (D) remains relatively rough, the milled surface (E) appears smoothest, and the 3D‐printed surface (F) shows minor residual lines.

The SEM images at 1000× magnification, as shown in Figure 3, illustrate the surface characteristics of unpolished and polished sides of three occlusal splint materials. The unpolished HC (Figure 3A) exhibits the highest roughness with prominent grooves, while the ML (Figure 3B) and 3D (Figure 3C) appear progressively smoother. After polishing, the HC surface (Figure 3D) remains relatively rough, the ML surface (Figure 3E) becomes the smoothest, and the 3D surface (Figure 3F) shows slight residual lines. These SEM images support the surface roughness data and highlight the differences in surface quality and polishability among the materials.

### 3.2. Microhardness Analysis

**Figure 4:**
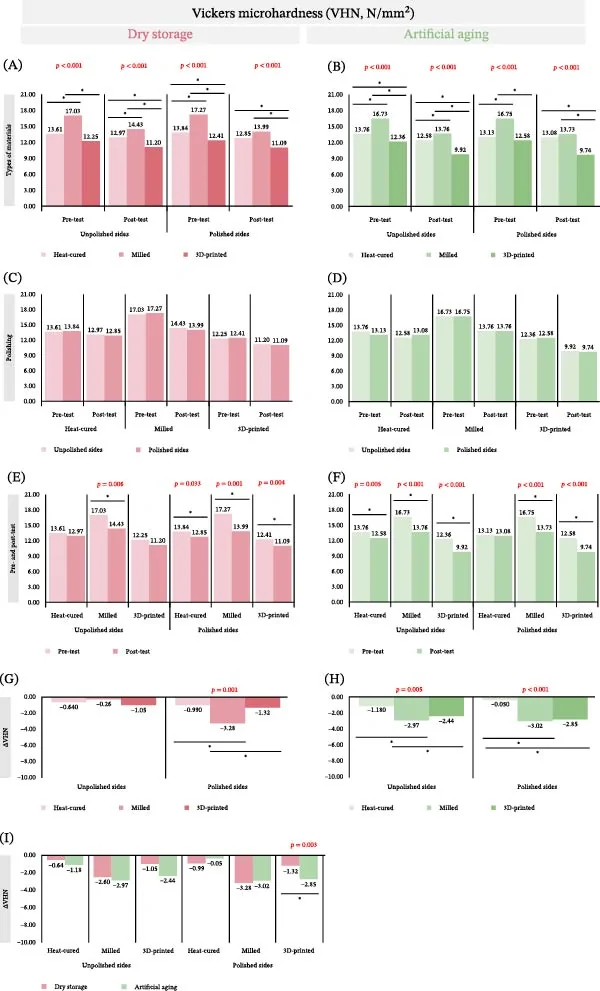
Vickers microhardness (VHN, N/mm^2^) of occlusal splint materials under different aging conditions. (A, B) Comparison of Vickers microhardness among heat‐cured, milled, and 3D‐printed materials on unpolished and polished sides under (A) dry storage and (B) artificial aging conditions. (C, D) Effect of polishing on microhardness within each material type under (C) dry storage and (D) artificial aging. (E, F) Comparison between pre‐test and post‐test Vickers microhardness values for each material and surface condition under (E) dry storage and (F) artificial aging. (G, H) Changes in Vickers microhardness (ΔVHN) after testing for each material on unpolished and polished sides under (G) dry storage and (H) artificial aging. (I) Overall comparison of ΔVHN between dry storage and artificial aging conditions across all materials and surface treatments. Bars represent mean VHN values (N/mm^2^). Asterisks ( ^∗^) indicate statistically significant differences (*p* < 0.05), and exact *p*‐values are provided, where applicable.

Figure 4 presents the mean Vickers microhardness values of the three types of occlusal splint materials. Significant differences in microhardness were observed among the materials (*p* < 0.05) at both pre‐test and post‐test under dry storage (Figure 4A) and artificial aging conditions (Figure 4B). The ML material exhibited the highest microhardness values under both conditions, whereas the 3D material showed the lowest values, particularly after artificial aging. Although most materials demonstrated a reduction in microhardness after aging, the ML material consistently maintained the highest value compared to that of HC and ML materials (Figure 4A,B).

The mean microhardness values between unpolished and polished surfaces were further compared (Figure 4C,D). Slight differences were observed between the two surface conditions for all materials; however, these differences were not statistically significant (*p* > 0.05).

Changes in microhardness (ΔVHN) were also evaluated (Figure 4G–I). All ΔVHN values were negative, indicating a reduction in microhardness after aging. Dry storage primarily affected the polished surfaces, particularly in the ML group (Figure 4G). In contrast, artificial aging influenced both unpolished and polished surfaces (Figure 4H). Overall, artificial aging resulted in a greater reduction in microhardness compared with dry storage, with a statistically significant decrease observed in the polished 3D specimens (Figure 4I).

### 3.3. Color Stability Analysis

**Figure 5:**
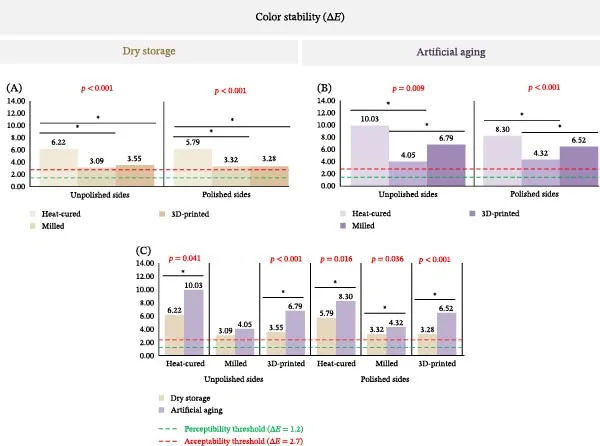
Color stability (Δ*E*) of occlusal splint materials under different aging conditions. (A, B) Comparison of color change (Δ*E*) among heat‐cured, milled, and 3D‐printed materials on unpolished and polished sides under (A) dry storage and (B) artificial aging conditions. (C) Overall comparison of Δ*E* values between dry storage and artificial aging across all materials and surface conditions (unpolished and polished sides). Bars represent mean Δ*E* values. The green dashed line indicates the perceptibility threshold (Δ*E* = 1.2), and the red dashed line indicates the acceptability threshold (Δ*E* = 2.7). Asterisks ( ^∗^) denote statistically significant differences (*p* < 0.05), and exact *p*‐values are provided, where applicable.

Figure 5 presents the mean color stability values (Δ*E*) of the three types of occlusal splint materials. Significant differences in color stability were observed among the materials (*p* < 0.05) under both dry storage (Figure 5A) and artificial aging conditions (Figure 5B). The HC material exhibited the greatest color change compared with the other materials, particularly after artificial aging. The effects of aging conditions on color stability were further compared (Figure 5C). Artificial aging resulted in more pronounced color changes than dry storage for all materials (*p* < 0.05), except for the unpolished ML specimens, which showed comparable Δ*E* values (*p* > 0.05).

From a clinical perspective, all materials demonstrated Δ*E* values exceeding the commonly accepted perceptibility and acceptability thresholds (Δ*E* > 2.7), particularly after artificial aging (Figure 5A–C). This finding suggests that color changes in these materials may be clinically noticeable and potentially unacceptable, especially in esthetically demanding situations.

Table 3 presents the results of the two‐way ANOVA evaluating the effects of material type, aging condition, and their interaction on changes in surface roughness, Vickers microhardness, and color stability.

**Table 3:** Two‐way ANOVA results for the effects of material type (material) and aging condition (aging), and their interaction (material × aging), on changes in surface roughness (ΔRa), Vickers microhardness (ΔVHN), and color stability (Δ*E*).

Variables	Sides	Source of variation	Type III SS	df	MS	*F*‐value	*p*‐Value
Changes in surface roughness (∆Ra)	Unpolished	Material	0.00309	2	0.00154	1.299	0.288
		Aging	0.00003	1	0.00003	0.023	0.881
		Material × aging	0.00039	2	0.00020	0.165	0.849
		*R* ^2^ = 0.090 (adjusted *R* ^2^ = −0.062)					
	Polished	Material	0.00003	2	0.00001	0.035	0.966
		Aging	0.00030	1	0.00030	0.786	0.382
		Material × aging	0.00092	2	0.00046	1.193	0.317
		*R* ^2^ = 0.098 (adjusted *R* ^2^ = −0.053)					

Changes in Vickers microhardness (∆VHN)	Unpolished	Material	21.200	2	10.600	7.384	0.002 ^∗^
		Aging	5.252	1	5.252	3.658	0.065
		Material × aging	1.771	2	0.885	0.617	0.546
		*R* ^2^ = 0.396 (adjusted *R* ^2^ = 0.295)					
	Polished	Material	42.138	2	21.069	23.539	<0.001 ^∗^
		Aging	0.110	1	0.110	0.123	0.728
		Material × aging	9.728	2	4.864	5.434	0.010 ^∗^
		*R* ^2^ = 0.659 (adjusted *R* ^2^ = 0.603)					

Changes in color stability (∆*E*)	Unpolished	Material	128.233	2	64.117	14.583	<0.001 ^∗^
		Aging	64.080	1	64.080	14.575	<0.001 ^∗^
		Material × aging	13.686	2	6.843	1.556	0.227
		*R* ^2^ = 0.610 (adjusted *R* ^2^ = 0.545)					
	Polished	Material	64.451	2	32.225	30.606	<0.001 ^∗^
		Aging	45.563	1	45.563	43.273	<0.001 ^∗^
		Material × aging	7.891	2	3.945	3.747	0.035 ^∗^
		*R* ^2^ = 0.789 (adjusted *R* ^2^ = 0.753)					

*Note*:  ^∗^Significant difference (*p* < 0.05).

Abbreviations: df, degree of freedom; MS, mean square; SS, sum of squares.

For surface roughness, neither material type (*p* = 0.288) nor aging condition (*p* = 0.881) significantly affected ΔRa on the unpolished surfaces. Similarly, on the polished surfaces, no significant effects were observed for material type (*p* = 0.955) or aging condition (*p* = 0.382). Although Figure 2G–I illustrates variations in ΔRa among materials and aging conditions, these differences were not statistically significant.

Changes in Vickers microhardness were also analyzed using two‐way ANOVA. On the unpolished surfaces, material type had a significant effect on ΔVHN (*p* = 0.002). Likewise, on the polished surfaces, material type significantly influenced microhardness changes (*p* < 0.001). In addition, a significant interaction between material type and aging condition was observed on the polished surfaces (*p* = 0.010), indicating that the effect of aging on microhardness depended on the material type. As shown in Figure 4I, only the 3D material exhibited a statistically significant change in ΔVHN following artificial aging.

For color stability, Table 3 shows that both material type and aging condition had significant effects on the unpolished surfaces (*p* < 0.001 for both). Similarly, on the polished surfaces, both factors were also significant (*p* < 0.001). Furthermore, a significant interaction between material type and aging condition was observed on the polished surfaces (*p* = 0.035).

## 4. Discussion

This study evaluated the surface roughness, microhardness, and color stability of three occlusal splint materials before and after aging. To compare the effects of dry storage and artificial aging, statistical analysis showed no significant differences in ∆Ra (Figure 2I), a significant difference in ∆VHN for only 1 of the 6 material groups (Figure 4I), and significant differences in ∆*E* for 5 of the 6 groups (Figure 5C). Based on these findings, the null hypothesis was accepted for surface roughness but rejected for microhardness and color stability under different aging conditions. Moreover, the statistical analyses revealed notable variations in measured values between materials fabricated using conventional and CAD–CAM methods. These results align with prior studies, such as Gibreel et al. [21] and Berli et al. [23].

### 4.1. Surface Roughness

The surface roughness analysis revealed that the 3D‐printed materials (MED610) had the lowest roughness on the unpolished surfaces and demonstrated minimal changes in roughness after aging. These findings indicated that 3D‐printing technologies produce smoother surfaces, which are consistent with Raffaini et al. [24] and Grymak et al. [25], who noted favorable surface characteristics in 3D‐printed dental materials. After polishing, the ML material (Smile‐Cam PMMA calcinable) exhibited the lowest roughness on the polished sides, while only the MED610 materials showed an increase in roughness after polishing. This finding is supported by the study of Grymak et al. [25], which demonstrated that 3D‐printing angles significantly influence surface roughness, with most splints exhibiting the lowest roughness at a 0° angle and some at 45°. Polishing was shown to reduce roughness in surfaces with high initial roughness but increase it in already smooth surfaces [25]. Similarly, in the present study, the 0° printing orientation resulted in the lowest roughness on unpolished surfaces, while polishing increased the roughness of these initially smooth 3D‐printed splints—likely due to the layered microstructure formed at the 0° angle, which may respond unevenly to polishing and altering the surface texture [5]. Hence, this suggests that 3D‐printed materials may not require a polishing procedure after fabrication if they initially possess a smooth surface.

Moreover, the present study provided data on both the unpolished and polished sides; the former represents the tissue surface contacting the patient’s teeth, and the latter represents the polished surface interacting with the oral mucosa and opposing teeth. Surface roughness values are linked to bacterial accumulation; lower roughness generally results in reduced microbial adhesion [9]. However, some studies have reported variability in bacterial and fungal adhesion depending on surface roughness [26, 27]. Therefore, occlusal splints may exhibit differing levels of microbial adhesion across their surfaces. Nevertheless, Bollen et al. [28] proposed a surface roughness threshold of 0.2 µm for bacterial colonization. In the present study, only the unpolished surfaces of HC occlusal splints exceeded this threshold. In contrast, the CAD–CAM occlusal splints exhibited significantly lower surface roughness compared to conventional splints. This suggests that CAD–CAM occlusal splints may present a lower risk of bacterial accumulation due to their smoother surface texture [18].

### 4.2. Microhardness

The microhardness analysis revealed that the conventional and CAD–CAM materials experienced a reduction in microhardness after dry storage and artificial aging. Although the ML occlusal splints (Smile‐Cam PMMA Calcinable) exhibited the highest reduction, they had the highest microhardness after the interventions. This may be because the ML occlusal splints are often created from clear PMMA blanks that have undergone controlled polymerization by the manufacturer before the milling procedure. The superior hardness of the ML material suggests their potential for better long‐term performance in clinical applications. These findings align with Berli et al. [23], who found that ML materials maintained higher microhardness compared with other fabrication methods.

Conversely, the 3D‐printed materials (MED610) had the lowest microhardness, particularly after undergoing artificial aging. In a similar finding, Prpic et al. [29] reported that 3D‐printed occlusal splints had the lowest microhardness, ranging from 4.88–11.29 N/mm^2^, compared with conventional and ML materials. This is due to the composition of the materials used in the printing technology, which must be semi‐solid, printable, biocompatible, and post‐cured, resulting in lower microhardness compared with PMMA [30]. On the other hand, after 30 days in different aging conditions, the HC occlusal splint (Meliodent heat cure) showed a trend of less change in microhardness compared with the ML and 3D‐printed materials. This indicates that the conventional occlusal splint may have relatively constant microhardness after long‐term use, aligning with Gibreel et al. [31] and Weżgowiec et al. [32].

Additionally, the microhardness of the occlusal splints was lower than that of dentin and enamel, which have Vickers hardness values of 65.6 and 274.8 N/mm^2^, respectively [33]. Therefore, when choosing the fabrication method for personalized occlusal splints, factors—such as microhardness values, the potential reduction in microhardness over time, and the possibility of enamel loss or dentin exposure in the patient’s teeth—should be considered.

### 4.3. Color Stability

There were significant changes in color stability among the three types of materials. Moreover, artificial aging conditions had a more pronounced effect on color stability than that of dry storage. These results suggest that wearing an occlusal splint in the oral cavity negatively affects its color stability. Furthermore, HC occlusal splints (Meliodent heat cure) exhibited the greatest change, especially under artificial aging, which could affect their transparency, cleanliness, and esthetics. When stored in dry conditions without additional factors, occlusal splints still showed changes in color stability. In real‐life scenarios, various methods are used to store occlusal splints, such as soaking in water, chlorhexidine solution, and denture cleanser, all of which warrant further study.

Although occlusal splint materials may undergo color changes, their clinical relevance must be considered. Paravina et al. [34] established a perceptibility threshold of ∆*E* = 1.2 and an acceptability threshold of ∆*E* = 2.7. According to these criteria, all occlusal splints in this study exhibited discoloration beyond the acceptability threshold, indicating the potential for noticeable color changes during use, even under dry storage conditions. Among the materials, the ML splint (Smile‐Cam PMMA Calcinable) demonstrated the lowest ∆*E* values in both dry storage and artificial aging. This minimal discoloration may be attributed to variations in chemical reactivity and polymerization [35], with ML materials likely benefiting from a higher degree of polymerization achieved during manufacturing.

### 4.4. Clinical Implications

The study’s findings have practical applications in dentistry, particularly in the selection of occlusal splint materials and the appropriate manufacturing methods to ensure that these splints possess suitable properties for various oral conditions (Table 4). Understanding the differences in roughness, microhardness, and color stability can help clinicians predict the amount of bacterial accumulation, durability, and long‐term color satisfaction when patients use their occlusal splints. These insights contribute to developing comprehensive data on material suitability for dental patients with diverse oral conditions and their biocompatibility [36].

**Table 4:** Summary of study findings and clinical recommendations.

Variables	Heat‐cured (conventional) Meliodent heat cure	Milled (CAD–CAM) smile‐Cam PMMA calcinable	3D‐printed (CAD–CAM) MED610
Surface roughness	High (especially unpolished); requires polishing	Low	Low; inherently smoother
Polishability	Requires thorough polishing after fabrication and adjustment	High polishability; smooth, glossy finish	Minimal polishing needed
Microhardness	Moderate; lower than milled	Highest; maintains hardness after aging	Lowest; reduced further with aging
Color stability	Most discoloration over time	Least discoloration	Moderate discoloration; better than conventional
Recommended use	Suitable for general use if polished well; not ideal for patients requiring high esthetics or wear resistance	Preferred for long‐term use, high bite force patients, and those needing durability and esthetics	Best for short‐term or interim use; not recommended for high occlusal loads or bruxism

Conventional Meliodent heat cure splints have high surface roughness, especially on unpolished areas, requiring thorough polishing after fabrication to minimize plaque accumulation and support hygiene. In contrast, CAD–CAM splints, both ML (Smile‐Cam PMMA Calcinable) and 3D‐printed (MED610), generally show lower roughness. ML splints can be polished to a high shine, while 3D‐printed splints typically need minimal polishing [19]. ML splints also exhibit higher microhardness and better wear resistance than HC and 3D‐printed types [37], even after artificial aging. The 3D‐printed splints show the lowest hardness, potentially limiting their durability in patients with high bite forces. Material selection should consider hardness, antagonist type, longevity, bite force, retention, and fabrication accuracy [37–42]. ML splints exhibited the least discoloration; however, all materials demonstrated some degree of color change over time, highlighting the need for regular monitoring or replacement.

### 4.5. Study Limitations

The present study has several limitations that should be considered when interpreting the findings. First, only one representative material from each fabrication category (HC, ML, and 3D‐printed) was evaluated. Consequently, the results should not be generalized to all commercially available occlusal splint materials. Contemporary occlusal splints are manufactured from a wide range of polymer systems, including heat‐ and self‐cured PMMA, thermoformed polymers, thermoformed materials combined with light‐cured build‐up resins, pre‐polymerized ML PMMA, stereolithography (SLA), digital light processing (DLP), and polyJet 3D‐printing resins, as well as urethane dimethacrylate (UDMA)‐based materials [5, 43]. These materials differ substantially in their polymerization characteristics, residual monomer content, water sorption, surface roughness, microhardness, wear resistance, color stability, flexural behavior, and biocompatibility, all of which may influence their clinical performance. Therefore, caution should be exercised when extrapolating the present findings to other materials or manufacturing systems.

Second, the manufacturing parameters used for both additive and subtractive fabrication were standardized for this investigation. However, material properties produced by CAD/CAM technologies are known to be influenced by processing variables, including the printer or milling system, layer thickness, build orientation, support design, post‐processing procedures, and polymerization protocols [44]. Variations in these parameters may alter the physical and mechanical properties of the final occlusal splints and should therefore be considered when interpreting the results [44, 45].

Third, this was an in vitro study, and the experimental conditions cannot fully reproduce the complex oral environment [5]. During clinical use, occlusal splints are continuously exposed to saliva, fluctuating temperatures, pH changes, cyclic masticatory loading, oral biofilm, and repeated cleaning procedures, all of which may accelerate material degradation and influence surface characteristics, mechanical properties, and color stability [5, 20, 44]. Consequently, the present findings should be interpreted within the limitations inherent to laboratory investigations.

Fourth, the present study evaluated only short‐term material properties and did not investigate long‐term clinical performance or biological safety [16, 36]. Clinical success depends not only on favorable physical and mechanical characteristics but also on long‐term resistance to wear, fracture, fatigue, dimensional changes, and color alteration under intraoral conditions [43]. Moreover, the long‐term biocompatibility of polymer‐based materials may be influenced by the release of residual monomers or other leachable substances, which were not assessed in this study [36, 46].

Finally, it is important to acknowledge that this study was exploratory in nature and used a relatively small sample size. Therefore, the findings should be interpreted with caution, and further confirmatory studies using larger sample sizes are warranted to validate these results.

Future studies should include multiple representative materials from each fabrication category to improve the external validity and generalizability of the findings. More clinically relevant aging protocols, including thermal cycling, pH cycling, mechanical fatigue loading, and prolonged water storage, should also be incorporated to better simulate the oral environment [17, 45]. In addition to surface roughness, microhardness, and color stability, future investigations should evaluate comprehensive mechanical and biological properties, such as flexural strength, fracture toughness, wear resistance, fatigue behavior, residual monomer release, and cytotoxicity [16, 36, 46]. Long‐term clinical studies are also warranted to validate laboratory findings and establish evidence‐based recommendations for material selection in occlusal splint therapy.

## 5. Conclusion

Under the specific in vitro conditions of this study, conventional and CAD–CAM occlusal splint materials exhibited significant differences in surface roughness, microhardness, and color stability. Compared with conventional Meliodent heat cure splints, CAD–CAM materials demonstrated lower surface roughness and greater color stability, while Smile‐Cam PMMA calcinable splints exhibited the highest microhardness. Artificial aging significantly reduced the microhardness of the MED610 materials and adversely affected the color stability of all tested materials but had no significant effect on surface roughness. Overall, the Smile‐Cam PMMA Calcinable materials demonstrated the most favorable combination of surface roughness, microhardness, and color stability under the conditions evaluated. However, these findings should not be interpreted as evidence of long‐term clinical superiority, as confirmation requires further standardized mechanical, biological, and clinical investigations.

## Author Contributions

**Uthai Uma, Jirayu Chansri, Thitiphat Itthipornpaisan, and Trin Thahong:** conceptualization, methodology, writing – review and editing. **Jirayu Chansri, Thitiphat Itthipornpaisan, and Trin Thahong:** investigation. **Uthai Uma:** data curation, formal analysis, project administration, supervision, visualization, writing – original draft.

## Funding

This work was supported by the Dental Research Fund, Dental Research Project, Faculty of Dentistry, Chulalongkorn University, Bangkok, Thailand (Grant 3200502#10/2023).

## Disclosure

All authors have read and approved the final version of the manuscript. The corresponding author had full access to all of the data in this study and takes complete responsibility for the integrity of the data and the accuracy of the data analysis. This funding was not involved in the study design, data collection, data analysis, manuscript writing, and decision to submit the manuscript for publication.

## Ethics Statement

This was an in vitro laboratory study involving no human participants, human‐derived specimens, or animals; therefore, institutional ethical approval and informed consent were not required.

## Conflicts of Interest

The authors declare no conflicts of interest.

## Data Availability

The data that support the findings of this study are available from the corresponding author upon reasonable request.
